# Early Loaded Single Implant Reinforced Mandibular Overdenture

**DOI:** 10.1155/2016/4213753

**Published:** 2016-06-14

**Authors:** K. Nischal, R. Chowdhary

**Affiliations:** Department of Prosthodontics, RajaRajeswari Dental College and Hospital, Bangalore 560074, India

## Abstract

Rehabilitating atrophied mandible with two-implant supported denture is a common treatment modality for implant retained removable overdenture in mandible. This paper aims to design a treatment modality where single implant reinforced overdenture is fabricated for a severely atrophied mandibular ridge with early loading protocol. Results of studies have shown that a single implant mandibular overdenture significantly increases the satisfaction and quality of life of patients with edentulism. Midline fracture of the prosthesis is the most common complication related to single implant and two-implant retained mandibular overdentures. To manage such complication, a thin metal mesh is used to reinforce the overdenture and also to make the prostheses lighter and cost effective as compared to conventional cast metal framework.

## 1. Introduction

Edentulism is a chronic condition and therapy is palliative, aimed to improve function and quality of life [1]. According to a survey, approximately 7% of the patients are not able to wear their dentures at all due to severe atrophy of the alveolar bone and are considered as “Dental Cripples.” When an edentulous patient is rehabilitated with a conventional complete denture on compromised alveolar bone, it often results in denture soreness, poor retention and stability, low chewing efficiency, and difficulty in pronunciation [2].

A single implant supported mandibular overdenture significantly increases the satisfaction and quality of life of patients with edentulism [3]. Studies have shown that even a single implant can significantly increase the maximum bite force [4]. Single implant overdenture can be an alternative treatment modality, as it is cost effective and less invasive than 2-implant retained overdenture. However, overdenture has a high incidence of fracture of the acrylic resin base at the point of the implant [5]. In the past 30 years, Professor Brånemark's concept of initially unloaded and submerged implants for a period of time to promote osseointegration was necessary, but current studies have shown that the concept of immediate and early loading of single implant overdenture can be a clinically viable treatment option for completely edentulous patients [6].

The aim of this study is to present the fabrication of mandibular implant overdenture by using single dental implant and early loading protocol.

## 2. Clinical Report

A 50-year-old female patient visited our Dental College and Hospital with a chief complaint of loose, ill-fitting, and broken mandibular denture. She was wearing conventional maxillary and mandibular denture for the past 10 years. Clinical examination revealed a highly resorbed mandibular alveolar bone, which was later confirmed with an orthopantomograph. Radiographic examination showed that mandibular bone was atrophic and 10 mm of bone height was there, between the mental foramens for implant placement.

Amongst the various treatment alternatives suggested to the patient were the conventional complete dentures with option of implant supported removable overdenture with varying number of implants. Depending on the patient's expectation, cost consideration, and diagnostic information, the treatment chosen was metal mesh-reinforced upper and lower single implant with locator attachment.

**Figure 1:**
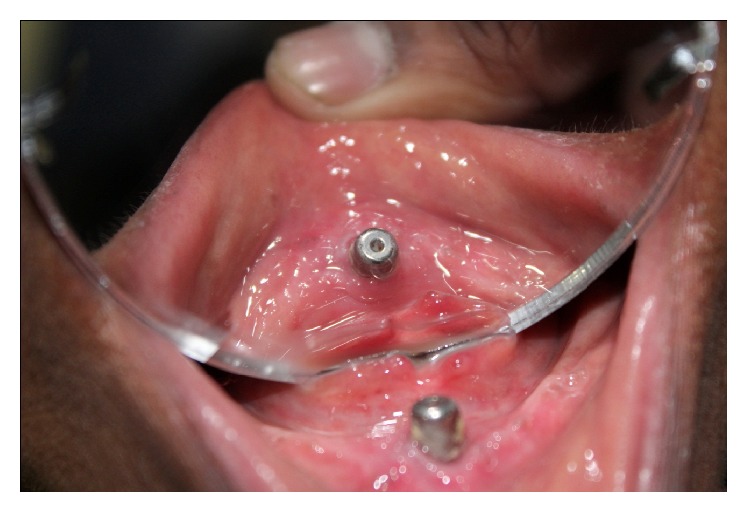
Healing after 6 weeks.

One root form implant (3.8 × 9.5 Myriad Implant System, Equinox Medical Technologies B.V., Netherlands) was placed into the parasymphyseal region of mandibular alveolar bone perpendicular to occlusal plane, after anaesthetizing the region with the local anesthetic agent (Lignospan Special, 2% lidocaine with 1 : 80,000 epinephrine, Septodont, France), and a mid crestal incision was made with relieving incision and the mucosa was reflected and the selected implant was placed after preparing the osteotomy as prescribed by the manufacturer. The implant achieved an insertion torque of 35 Ncm. The reflected flap was later sutured using vicryl (absorbable, polyglactin 910) suture. A postoperative radiograph was taken to confirm the position of the implant placement (Figure 1). Postoperative care instructions were given to the patient and medications were prescribed (Amoxicillin 500 mg TDS, Tinidazole 500 mg BD, and Ibuprofen 200 mg TDS for 5 days). After a week from the surgery, the surgical site was evaluated for any infection and discharge and when it was found that the healing was appropriate and the sutures were removed. The implant was allowed to heal for 6 weeks.

## 3. Procedure

**Figure 2:**
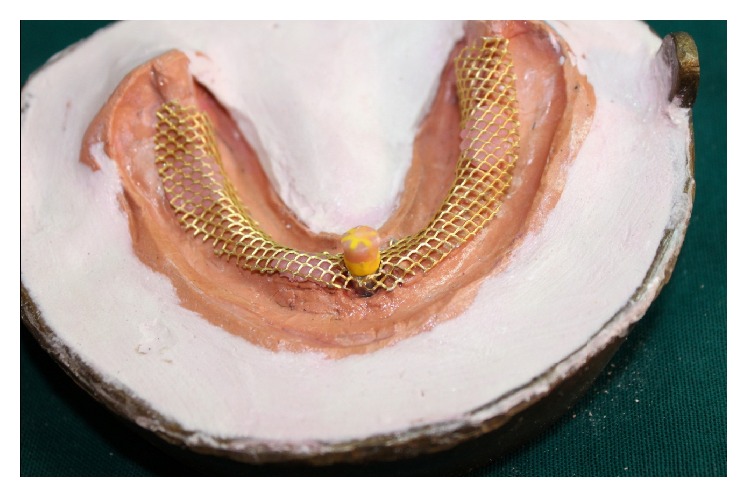
Metal mesh adapted on the master cast.

(1) After 6 weeks of healing, preliminary impression was made with irreversible hydrocolloid (alginate, ADA specification number 18) using rim lock perforated edentulous trays (ADA specification number 87), and the primary cast was poured with high strength dental stone (Type III).

(2) Over the primary casts, wax spacers were adapted, and custom trays were fabricated using self-cure acrylic resin (ADA specification number 139).

(3) Custom trays were border-moulded with addition silicone (polyvinyl siloxane by 3 m, ADA specification number 19) and a pick up impression using closed tray technique was made.

(4) Locator implant analog was attached to the impression coping and secondary impression was poured with high strength dental stone (Type III).

(5) Resin record bases were fabricated with the transfer coping as a guide for accurate position of locator housing in the final denture base.

(6) Wax occlusal rims (modelling wax) were fabricated to average dimension, and jaw relation was recorded.

(7) Bilateral occlusion was achieved during teeth arrangement.

(8) Assessment of the trial dentures was done clinically. Aesthetics were accepted by the patients.

(9) Trial dentures were waxed up and flasking and dewaxing were carried out in the same fashion.

(10) Additional retentive grooves were given on the acrylic teeth (ADA specification number 15) after dewaxing.

(11) Metal mesh (Bredent USA, metal mesh, 0.4 mm thick) was adapted on the secondary casts after dewaxing procedure, and three drops of self-cure acrylic resin were used as stops and applied to the model (Figure 2).

(12) The reinforced mesh was positioned and the resin is allowed to harden as long as the resin has a highly viscous consistency. If reinforcing mesh does not adhere to the model prior to pressing, in that case a cyanoacrylate adhesive can be used, and stops created a 0.5 to 1 mm space between mesh and tissue surface which helped the heat cure resin to flow through the pores and fill the space which makes mesh completely embedded inside the denture resin.

(13) After refining the contour, it was packed and processed with heat cure polymerized resin (Lucitone, Dentsply, USA).

(14) The dentures were then finished and polished.

(15) The yellow transfer coping was still present in the denture which was trimmed and the final locator (Zest Anchor, United States) female housing was picked up in the denture from that position with the help of self-cure acrylic resin and patient was instructed to bite in centric relation.

(16) The black nylon processing ring from the male housing was removed with an appropriate tool and blue dual retention locator male processing ring was inserted as per patient's expectation of amount of retention required (Figure 3).

(17) Posttreatment therapy included 24 hrs, 1 week, and 6 weeks of evaluation involving evaluation of occlusion, oral hygiene, and comfort (Figure 4).

(18) No posttreatment complications were seen and patient was followed up every six months for 2 years.

## 4. Discussion

The two-implant supported overdenture has been a very popular treatment option and has been widely accepted [7]. Recent studies done by Harder et al. in 2011 have also shown that single implant overdenture is equally suitable treatment option for patients for whom cost consideration is an issue of concern [8]. Single implant overdenture has significantly improved the quality of life, retention, efficiency of chewing, phonetics, and patient's social life. According to a study done by Gonda et al. in 2007, the single implant in overdenture becomes the fulcrum and the denture base area around the implant is usually thin so the overdenture is susceptible to fracture. So reinforcement can effectively reduce the strain and prevent the deformation of the overdenture [9]. A 3D finite element analysis done by Liu et al. in 2013 showed that single implant retained mandibular overdenture does not show any damaging strain concentration in the bone around an implant because when vertical load is applied on the implant overdenture, it rotated side to side but under same loading conditions. Two-implant retained mandibular overdenture showed more apparent rotations around the fulcrum line passing through the two implants and the maximum equivalent stress in the abutments was higher in the other models [10]. A study was done by Chen et al. in 2011, which showed that locator attachment had a greater freedom of rotation than O-ring attachment. Amount of stress magnitude being transferred to implants depends on the degree of rotation of the attachment system. Rotation within acceptable limits can reduce the stress concentration on dental implants and prevents the crestal bone loss [11]. In another study done by Cakarer et al. in 2011, they reported no difference between ball attachment and locator systems regarding implant failure, replacement of attachments, and fracture of overdentures [12]. They found that the advantages of locator attachment were more compared to a ball or bar clip attachment. In a study done by Cehreli in 2010, it was shown that prosthetic maintenance requirements for implant overdentures like dislodgement, worn or loose matrix, or its respective housing were more common in ball attachment after the first year of loading [13]. In another study done by Sun et al. in 2014, it was shown that a single implant retained mandibular overdenture can significantly improve the masticatory efficiency (ME) and Oral Health Related Quality of Life (OHRQoF) and improvement in OHRQoF is mainly because of improved ME and also improved chewing efficiency and pain relief contributes to significant improvement of OHRQoF [14]. According to the definition by third ITI consensus conference held in 2003 in Gstaad, Switzerland, of early loading protocol, and a study done by El-Sheikh et al. in 2012, 1-year preliminary results indicated that early loading of single chemically modified surface implant used to retain a mucosa borne mandibular overdenture is a safe, reliable, and cost effective treatment [15]. Mini implants have also been suggested as an alternative with advantages such as less invasive and lower costs [16]. However narrow and mini implants used for overdenture should have at least 10 mm length, in relation to their diameter, but also to bone height. According to a study done by De Souza et al. in 2015, overdentures retained by 4 or 2 mini implants can achieve OHRQoL and satisfaction at least comparable with that of 2 standard implants. However, the survival rate of mini implants is not as high as that of standard implants [17]. In a finite element analysis conducted by Chang et al. in 2016 on mechanical response comparison in an implant overdenture retained by ball attachments on conventional regular and mini dental implants, they concluded that overdentures retained using ball attachments on mini dental implants in poor edentulous bone structure increase the surrounding bone strain over the critical value, thereby damaging the bone when compared to the regular diameter implant [18]. Elsyad in a study showed that mini implants retained mandibular overdenture required a considerable amount of prosthetic maintenance and repair over a period of time [19]. When it comes to loading the mini dental implants, Maryod et al. specified that immediate and early loading protocols showed good clinical results with favorable peri-implant tissue response 3 years after implant insertion [20]. However a study done by Šćepanović et al. in 2015 stated that 1-year bone resorption around immediately loaded mini dental implants is within the clinically acceptable range for standard implants [21]. According to Carl E. Misch prosthetic classification, RP5 prosthesis is subjected to more bone loss posteriorly in comparison to RP4 prosthesis. Therefore a single implant overdenture needs to be relined over a period of time for better prognosis in the future.

## 5. Conclusion

Within the limitations of this study, it appears that early loaded single implant overdenture reinforced with metal mesh is reliable treatment option in prosthetically maladaptive edentulous patients and patients for whom cost is a major issue of concern; it can provide a beneficial outlay over a 2-year observation period.

## Figures and Tables

**Figure 3:**
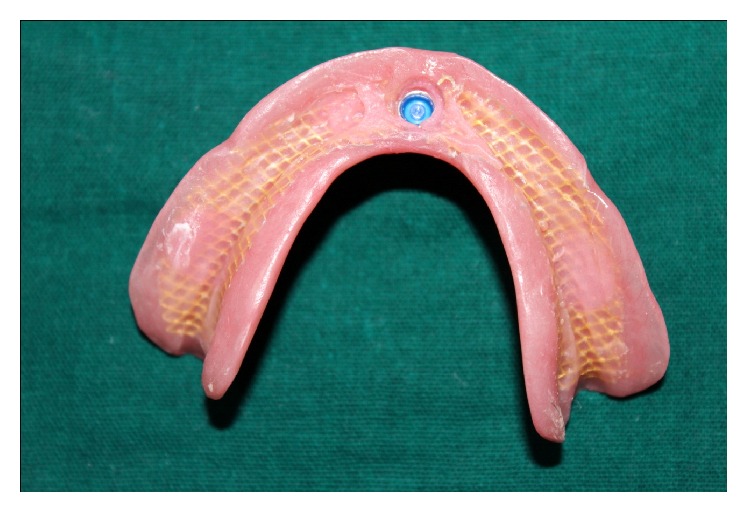
Female housing picked up in the denture.

**Figure 4:**
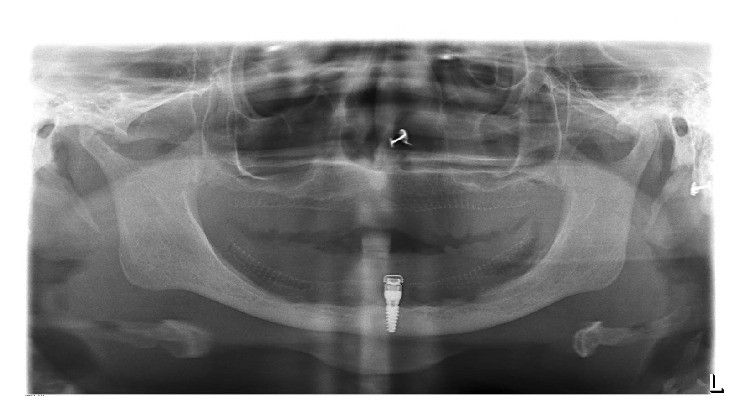
Postprosthesis opg.
